# Assessment of the Relation Between Asthma Severity and Serum Vitamin D Levels: A Cross-Sectional Study

**DOI:** 10.7759/cureus.46826

**Published:** 2023-10-11

**Authors:** Navjot Kaur, Vipul Kumar, Jagjot Singh, Hritvik Jain, Paras Paras, Nirmaljeet Kaur, Ashwani K Sareen

**Affiliations:** 1 Department of Pediatrics, Government Medical College, Amritsar, Amritsar, IND; 2 Department of Pediatrics, All India Institute of Medical Sciences, Jodhpur, Jodhpur, IND; 3 Department of Pediatrics, Government Medical College, Patiala, Patiala, IND

**Keywords:** asthma diagnosis, acute asthma managment, asthma exacerbations, 25-oh vitamin d, vitamin-d deficiency, pediatric asthma, gina guidelines

## Abstract

Introduction

Vitamin D3’s importance for bone health in children and its potential role beyond musculocutaneous health is an ongoing area of research. This study assesses vitamin D3 deficiency prevalence in asthmatic children and its correlation with asthma cases and healthy controls.

Methods

This cross-sectional study was conducted in a tertiary care hospital in Punjab, India among children between 5 and 15 years of age. Fifty children diagnosed with “bronchial asthma” who were under follow-up in the asthma clinic in outpatient and inpatient patients were enrolled as cases. Age-matched 50 healthy controls who presented for routine check-ups were enrolled in the control group. Demographic details were noted and clinical examination was done in all the cases. 25-(OH) vitamin D levels were estimated and compared in all cases and controls. The study also analyzed the relationship between 25-(OH) vitamin D levels with asthma control and severity.

Results

The study showed that serum vitamin D3 level was significantly decreased in asthmatic children (24.62 ± 14.95 ng/ml) as compared with the healthy control group (32.08 ± 12.22 ng/ml). Also, serum vitamin D3 level was significantly decreased in children with uncontrolled asthma (12.06 ± 4.68 ng/ml) as compared to children with well-controlled asthma (44.82 ± 10.48 ng/ml).

Conclusion

The findings showed that low serum levels were observed more in asthmatic children as compared to healthy children. A correlation was also found between vitamin D3 levels and asthma severity, its control, and the number of acute exacerbations in the last year.

## Introduction

Bronchial asthma is a common chronic inflammatory disease of the airways in children [1]. It is characterized by increased inflammation of the airways, airway hyperresponsiveness, and obstruction to airflow in response to specific triggers leading to recurrent attacks of wheezing, difficulty in breathing, heaviness in the chest, and cough. These episodes are usually associated with a bronchial constriction that either resolves spontaneously or after medication. The increasing prevalence of asthma may be attributed to various etiological and environmental factors such as air pollution, dietary modifications, allergens, family history of atopy or asthma, and lifestyle changes [1, 2].

The prevalence of bronchial asthma is very high in industrialized nations [3, 4]. Nowadays, the number of children with asthma is rising [4]. In 2001, 8.7% of children had asthma and if this trend continues, the CDC predicted that 400 million people would have asthma by 2025 [5]. A step-up approach has been suggested for asthma pharmacotherapy, which involves a variety of management modalities. Recent studies observed that vitamin D deficiency has been linked to various chronic diseases including asthma [6]. Vitamin D receptors (VDRs) are present in various cells of the immune system like macrophages, dendritic cells, monocytes, and activated B and T cells [7]. Hence vitamin D plays an important role in immunomodulation in the body [8]. Both inflammation and immune system modulation of the respiratory epithelium are regulated by a number of genes whose transcription is controlled by VDRs [9]. Interleukin-17 (IL-17) and IL-13 are pro-inflammatory cytokines that are suppressed by vitamin D, while anti-inflammatory cytokines like IL-10 are promoted. Also, vitamin D changes the balance of T cell response from the Th1 phenotype to the Th2 phenotype [10, 11].

Many clinical researches are being conducted around the world to study the novel role of vitamin D in non-musculocutaneous health. Although systematic reviews and meta-analyses are lacking, there is rising evidence linking vitamin D deficiency to childhood asthma. With regard to the existing controversies, we performed this study to evaluate the prevalence of vitamin D deficiency in asthmatic children and to study the correlation of vitamin D levels with asthma control, severity of asthma, and number of exacerbations in the last year.

## Materials and methods

This was a hospital-based cross-sectional study conducted in the Department of Pediatrics, Government Medical College, Amritsar, Punjab, India from December 2022 to January 2023. Fifty pediatric patients who visited the outpatient department or were admitted to the inpatient ward, with a diagnosis of bronchial asthma as per the Global Initiative for Asthma guidelines (GINA), were included in this study as cases based on inclusion and exclusion criteria. 50 healthy children of comparable age groups were enrolled as the control group. Informed written consent was obtained from parents or guardians of the children included in this study. Ethical approval and clearance were sought from the Institutional Ethics Committee before the initiation of the study (GMC/IEC/22/AK/82).

A semi-structured self-designed questionnaire was used to assess demographic details, clinical history, physical exam findings, and other co-morbidities. Past history and history of asthma in any family member were noted. Systemic examination was done in all the children and anyone found to have any major systemic illness was excluded from the study to avoid bias in the observed vitamin D levels. Controls were healthy children who presented for routine check-ups, had normal physical examinations, and did not demonstrate any symptoms of vitamin D deficiency. Nutritional status was classified as per the World Health Organization standards using BMI charts. The severity of asthma was categorized as per Global Initiative for Asthma guidelines.

Based on Global Initiative for Asthma guidelines, participants were categorized into four groups (Table 1). 

**Table 1:** Categorization of enrolled participants based on GINA guidelines. GINA: Global Initiative for Asthma

Category	Groups
1.	Intermittent asthma
2.	Mild persistent asthma
3.	Moderate persistent asthma
4.	Severe persistent asthma

Depending on the asthma control, participants were categorized into three groups (Table 2). 

**Table 2:** Categorization of enrolled participants based on the level of asthma control.

Category	Groups
1.	Well-controlled asthma
2.	Partly controlled asthma
3.	Uncontrolled asthma

An amount of 2.5 ml of blood sample was collected in plain vials. The serum was separated by centrifuging the sample at -4^o^C and was analyzed on the same day for the levels of 25-(OH) vitamin D3. The serum level was measured by the chemiluminescence method of estimation.

On the basis of serum vitamin D levels, all participants were divided into four groups (Table 3). 

**Table 3:** Categorization of enrolled participants based on serum vitamin D3 levels.

Category	Groups	Vitamin D3 levels
1.	Severely deficient group	< 10 ng/ml
2.	Deficient group	10-20 ng/ml
3.	Insufficient group	20-30 ng/ml
4.	Sufficient group	>30 ng/ml

Vitamin D3 levels were compared between asthmatic and non-asthmatic groups. Also, the correlation between asthma severity and vitamin D3 levels was assessed in the asthmatic group. Statistical analysis was done using Statistical Package for Social Sciences (SPSS) ver.21 software (IBM Corp., Armonk, USA). Chi-square test, t-test, and ANOVA were used for comparison between the two attributes and a p-value less than 0.05 ( p < 0.05) was taken as statistically significant.

## Results

This study was conducted to analyze vitamin D3 levels in children with bronchial asthma and compared it with vitamin D3 levels in healthy children. In this study, out of 50 asthmatic children, 27 were male and 23 were female. In the control group, out of 50 healthy children enrolled, 29 were male and 21 were female. No significant difference was observed on the basis of gender distribution in the asthmatic and control group with a p-value of 0.687 (p > 0.05). The mean age of children in the asthmatic group was 9.82 ± 2.92 years while it was 9.87 ± 2.49 years in the control group (minimum = 5 years, maximum = 15 years). The mean age of children in both groups was found to be comparable by using a t-test with a statistically insignificant p-value of 0.941 (p > 0.05) (Table 4). 

**Table 4:** Comparison of mean age and vitamin D3 levels in asthmatic and control groups. Age is represented as 'mean ± SD'. A p-value less than 0.05 (p < 0.05) is considered statistically significant.

Parameter	Asthma Group	Control Group	t-test (p-value)
Mean Age (in Years)	9.82 ± 2.92	9.87 ± 2.49	0.941
Mean Vitamin D3 levels (in ng/dl)	24.62 ± 14.95	32.08 ± 12.22	0.005

Similarly, the height, weight, and body mass index of the children in cases and control were found to be comparable and there was no statistically significant difference in these parameters in both the groups. The mean value of vitamin D3 was more in the control group (32.08 ± 12.22 ng/dl) as compared to the asthmatic group (24.62 ± 14.95 ng/dl). The dataset was analyzed using a t-test and a statistically significant difference was found in the mean values of vitamin D3 among studied groups with a p-value of 0.005 (p < 0.05) (Table 4).

**Figure 1:**
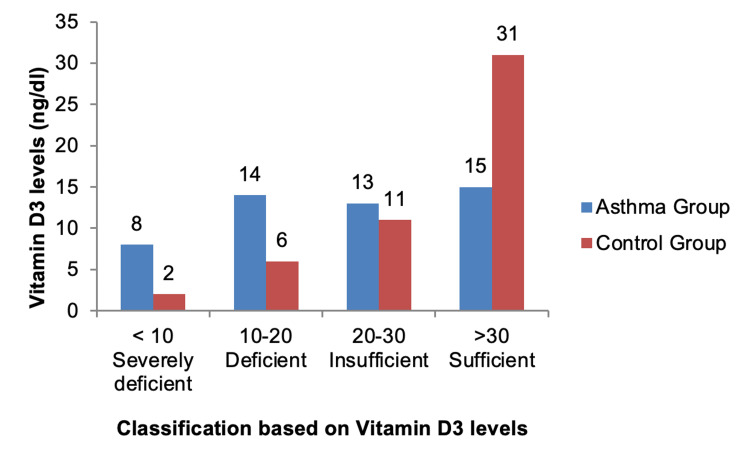
Comparison of serum vitamin D3 levels in asthmatic and control groups.

Sufficient levels of serum vitamin D3 (> 30 ng/dl) were found in 31 (62%) healthy children in the control group while only 15 (30%) children had sufficient levels of vitamin D3 in the asthmatic group. Similarly, children with severely deficient vitamin D3 levels (< 10 ng/dl) were eight (16%) in the asthmatic group as compared to two (4%) in the control group. Data analyzed using a chi-square test showed a significant difference in levels of vitamin D3 in both the studied groups with a highly significant p-value of 0.0057 (p < 0.05) (Figure 1).

**Figure 2:**
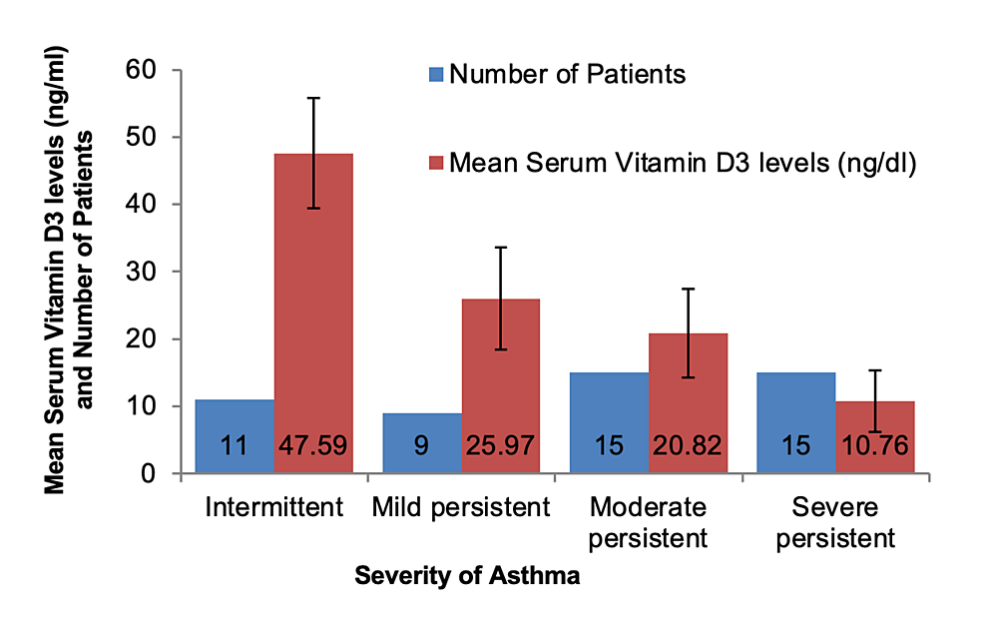
Correlation between mean serum vitamin D3 levels and severity of asthma.

GINA guidelines were used to assess the severity of asthma in children at the time of enrolment in the study. The mean value of serum vitamin D3 was more in children who had intermittent asthma (47.59 ± 8.20 ng/dl) as compared to children who had moderate persistent or severe persistent asthma with mean values 20.82 ± 6.55 ng/dl and 10.76 ± 4.60 ng/dl, respectively. One way ANOVA test was used to analyze the relationship between serum vitamin D3 levels and the severity of asthma which showed a statistically highly significant p-value of < 0.001 (Figure 2). 

GINA guidelines were used for the assessment of control of asthma. Our study showed that 13 (26%) had well-controlled asthma, 17 (34%) had partly controlled asthma, and 20 (40%) had uncontrolled asthma. A one-way ANOVA test was used to find the relationship between vitamin D3 levels and asthma control, and it showed that children with a high mean value of vitamin D3 (44.82 ± 10.48 ng/dl) had well-controlled asthma. Vitamin D3 levels were low in children with partly controlled and uncontrolled asthma with mean values of 23.94 ± 6.85 ng/dl and 12.06 ± 4.68 ng/dl respectively. One-way ANOVA was used to analyze the relationship between asthma control and vitamin D3 levels, which showed a highly significant p-value of < 0.001 (Table 5). 

**Table 5:** Comparison of serum vitamin D levels among well-controlled, partly controlled, and uncontrolled asthmatic children. 'N' denotes the number of children. Serum Vitamin D3 levels are reported in 'mean ± SD'. A p-value of less than 0.05 (p < 0.05) is considered statistically significant.

Control of asthma	Number (N)	Serum Vitamin D3 levels (ng/dl)	ANOVA (F critical value)	p-value
Well-controlled	13	44.82 ± 10.48	3.195	< 0.001
Partly controlled	17	23.94 ± 6.85		
Uncontrolled	20	12.06 ± 4.68		

The number of acute exacerbations of bronchial asthma (≥ 2) in the last year was higher in children with severe deficiency of vitamin D3 levels as compared to children who had sufficient vitamin D3 levels. Among the asthmatic group, five children required pediatric intensive care unit (PICU) admission, the rest were managed on an outpatient basis with metered-dose inhalers. Data were analyzed by chi-square test and it was concluded that children with low vitamin D3 levels had more episodes of acute exacerbation of bronchial asthma as compared to children with sufficient and insufficient vitamin D3 levels with a significant p-value of 0.000059 (p < 0.05) (Table 6). 

**Table 6:** Association between serum vitamin D3 levels and the number of acute exacerbations in the last one year. A p-value of less than 0.05 (p < 0.05) is considered statistically significant.

Number of exacerbations of bronchial asthma in the last one year	Serum Vitamin D3 levels (ng/dl)				p-value
	< 10 (Severely deficient)	10-20 (Deficient)	20-30 (Insufficient)	> 30 (Sufficient)	
>/= 2 episodes	7	13	7	2	0.000059
< 2 episodes	1	1	6	13	

## Discussion

Vitamin D3 deficiency is very common in children in developing countries [12]. Research is being done worldwide to find the role of Vitamin D3 in non-musculocutaneous diseases like asthma. In the present study, we enrolled 50 asthmatic children as cases and 50 healthy children of the same age group as the control. The mean value of vitamin D3 was higher in the control group (32.08 ± 12.22 ng/dl) as compared to the asthmatic group (24.62 ±14.95 ng/dl). 

Similar correlations between the severity of asthma and vitamin D levels have also been reported by Pragalatha et al [13] and Jat et al [14] in their study. Kranam et al [15] conducted a cross-sectional study of 60 children with asthma and concluded that hypovitaminosis D is frequent in children with asthma and is associated with exacerbations, decreased lung functions, and severe disease. A study conducted by Shebl et al [16] concluded that Vitamin D deficiency was highly prevalent in asthmatic patients which was similar to the results of our study. Our results were in concordance with a study conducted by Khan et al which also observed a similar correlation between vitamin D3 levels and asthma control [17].

Our study concluded that children with low vitamin D3 levels had more episodes of acute exacerbation of bronchial asthma as compared to children with sufficient and insufficient vitamin D3 levels with a significant p-value of 0.000059. Similar findings were demonstrated in a study conducted by Krishnan et al [18], which found that asthma exacerbation in terms of emergency room visits and acute-reliever asthma medication usage was further reduced by vitamin D supplementation. Randomized control trials provide some low-quality evidence to support vitamin D supplementation for the reduction of asthma exacerbations [19].

Our study had several limitations. Firstly, because our study was a single-center study, the results might not equate with other trials or with other multi-centric studies. Secondly, this study was based in India, a country where vitamin D deficiency is known to be higher than the general population prevalence, which confers a potential lack of generalisability in other countries and even might pose as a bias. We propose the idea of undertaking larger multi-centric and multinational clinical trials for accurately estimating this correlation. Thirdly, we did not check for a reduction in acute exacerbation of asthma and changes in the severity of asthma after supplementing vitamin D3 in these enrolled children. This limitation becomes particularly important when proposing a causation hypothesis between vitamin D3 deficiency and the severity of asthma. 

## Conclusions

As clinically observed, vitamin D3 deficiency is common in children. Low serum vitamin D levels are more commonly seen in asthmatic children as compared to healthy children. Asthmatic children with vitamin D deficiency have poorly controlled asthma and exhibit more acute exacerbations. Therefore vitamin D3 may be included in the regular treatment of asthmatic children. However, furthermore, studies are needed to see the effect of vitamin D3 supplementations on asthma control, its severity, and the number of acute exacerbations.
